# Effect of cavity shape on microstructural evolution of pure aluminum in electrically-assisted solidification

**DOI:** 10.1038/s41598-023-29522-y

**Published:** 2023-02-28

**Authors:** Seung Jun Choi, DongEung Kim, Moonwoo La, Moon-Jo Kim

**Affiliations:** 1 Smart Liquid Processing R&D Department, Korea Institute of Industrial Technology, Incheon, 21999 Republic of Korea; 2 School of Mechanical Engineering, Korea University of Technology and Education, Cheonan, 31253 Republic of Korea

**Keywords:** Computational methods, Metals and alloys, Mechanical properties

## Abstract

Grain refinement is a crucial issue in metallic materials. One of the emerging techniques to obtain equiaxed grains is to apply an electric current to the liquid metal during solidification. With this view, in this paper, the effect of electric current on the solidification behavior in various cavity shapes of mold was investigated. Cylinder-, cube-, and cuboid-shaped cavities designed to have similar cavity volume were used. By applying an electric current during the solidification of liquid aluminum, the grains were effectively refined with a grain size of approximately 350 µm for all three types of cavities. The circulating flow of liquid aluminum was observed to have a similar shear rate intensity in all three types of cavities, which is known to be sufficiently high (over hundreds of s^−1^) to induce dendrite fragmentation resulting newly generated nuclei. Dispersion of nuclei on unsolidified aluminum appeared differently according to the shape of the cavity, which influences final shape of refined zone. The area fraction of refined zone was affected by the relative relationship between the solidification completion time and the electric current application time. This study will provide insight to control of process parameters when electrically-assisted solidification is applied to a real product with a complex shape.

## Introduction

Control of microstructure is essential based on the understanding of solidification behavior in liquid metal processing to obtain high strength and good ductility^1–3^. The solidification behavior of liquid metal is influenced by various factors, such as composition, heat flow in the solidifying system, and quality of liquid metal. In particular, the cooling rate during solidification is a key parameter for determining the solidification structure^4,5^. For example, changing the size or shape of the cavity in mold can result in a different solidification structure, owing to a change in the cooling rate, even if the material has the exact same alloy composition. Consideration of thermal field gradient affecting solidification structure is also essential for the production of complex shape with various size of casting parts.

Various techniques, such as chemical additives^6–8^ and rapid cooling methods^9,10^ have been used to control the solidification structure in the casting industry. Chemical additives have been considered a common technique to refine or modify the phase. The rapid cooling method is also frequently adopted to obtain a fine solidification structure. However, the former has a few disadvantages, namely, fading additives, and undesirable formation of defects, such as pores and intermetallics^11,12^. The latter has limitations in increasing the cooling rate, depending on the mold material, product shape, and working environment. To overcome these drawbacks, treatment of liquid metal using external energy, including mechanical vibration^13–15^, electromagnetic stirring^16–19^, and ultrasonic vibration^20–22^, have been introduced, which can be alternative methods to obtain mechanical properties that meet the requirements of the end products. Recently, a casting method using an electric current as an external energy source has been proposed^23–26^. In this method, an electric current is applied directly to the liquid metal through the electrodes during solidification. Grain refinement or modification of the phase is known to be the main effect when electric current is applied to the liquid metal. In particular, grain refinement has been confirmed by many previous studies^27–29^ since 1985, when this technique was first reported^30^. In various metals, including Pb–Sn alloys^31–33^ and Cu–Bi–Sn alloys^34,35^, grains were effectively refined by applying an electric current during solidification. For example, the grain size of 1700 µm in the as-cast Sn–Bi alloy was decreased to approximately 400 µm by applying an electric current during solidification. Currently, research on grain refinement in aluminum alloys is attracting attention, owing to the growing demand for lightweight materials. The grain size of pure aluminum (Al)^27–29,36^ and α-Al in Al–Si alloys^37,38^ is significantly reduced by applying electric current during solidification. Raiger et al.^29^ reported that the grain size of pure aluminum decreased by approximately 82% with an application of an electric current, as compared to that without an application of an electric current.

Various hypotheses, including dendrite fragmentation and Joule heating effects, have been proposed to understand the effect of electric current on metal solidification. The dendrite fragmentation effect is frequently suggested as one of the main hypotheses for grain refinement by the application of an electric current. This hypothesis was proposed by researchers who confirmed the existence of the forced flow of liquid metal, caused by the Lorentz force, under an electric current through numerical simulation^28,29^. They reported that the forced flow of liquid metal due to the Lorentz force induced by the electric current could generate fragments of previously grown dendrites, leading to grain refinement by supplying additional nuclei. Wang et al.^39^ performed in-situ observations of the evolution of dendrite morphology during solidification under an electric current, based on the synchrotron radiation imaging technique. It was suggested that the dendrite morphology was modified by Joule heating at the dendrite tip. Li et al.^24^ also concluded that the current-induced Joule heating enhanced the nucleation rate, resulting in grain refinement in pure aluminum. However, the underlying mechanism of the effect of the electric current on the solidification structure remains controversial.

Although the underlying mechanism has not yet been clearly established, practical studies are required to consider this technique in real industry. Ma et al.^40^ investigated the solidification structure of pure aluminum with different electrode configurations. The position of the electrodes and the distance between the electrodes were set as variables. They found that the portion of refined grain area could be influenced by the position of the electrode. However, few studies have considered instrumental setup through systematic studies. To the best of our knowledge, no studies have considered the cavity shape of mold under an application of electric current during solidification.

This study aims to determine the correlation between the cavity shape and solidification structure under the application of an electric current during solidification. Pure aluminum was selected as the representative material to minimize various factors due to alloying elements. We prepared three types of cavities with different cavity shapes while fixing the volume of the cavity. The effect of electric current on grain refinement based on macro- and microstructural observation was analyzed. Numerical modeling based on fluid dynamics was also conducted by considering the effect of the electric current on liquid aluminum. Finally, the effect of electric current on the solidification behavior has been discussed, with a focus on the cavity shape, based on experimental and numerical approaches.

## Methods

### Experimental method

**Figure 1:**
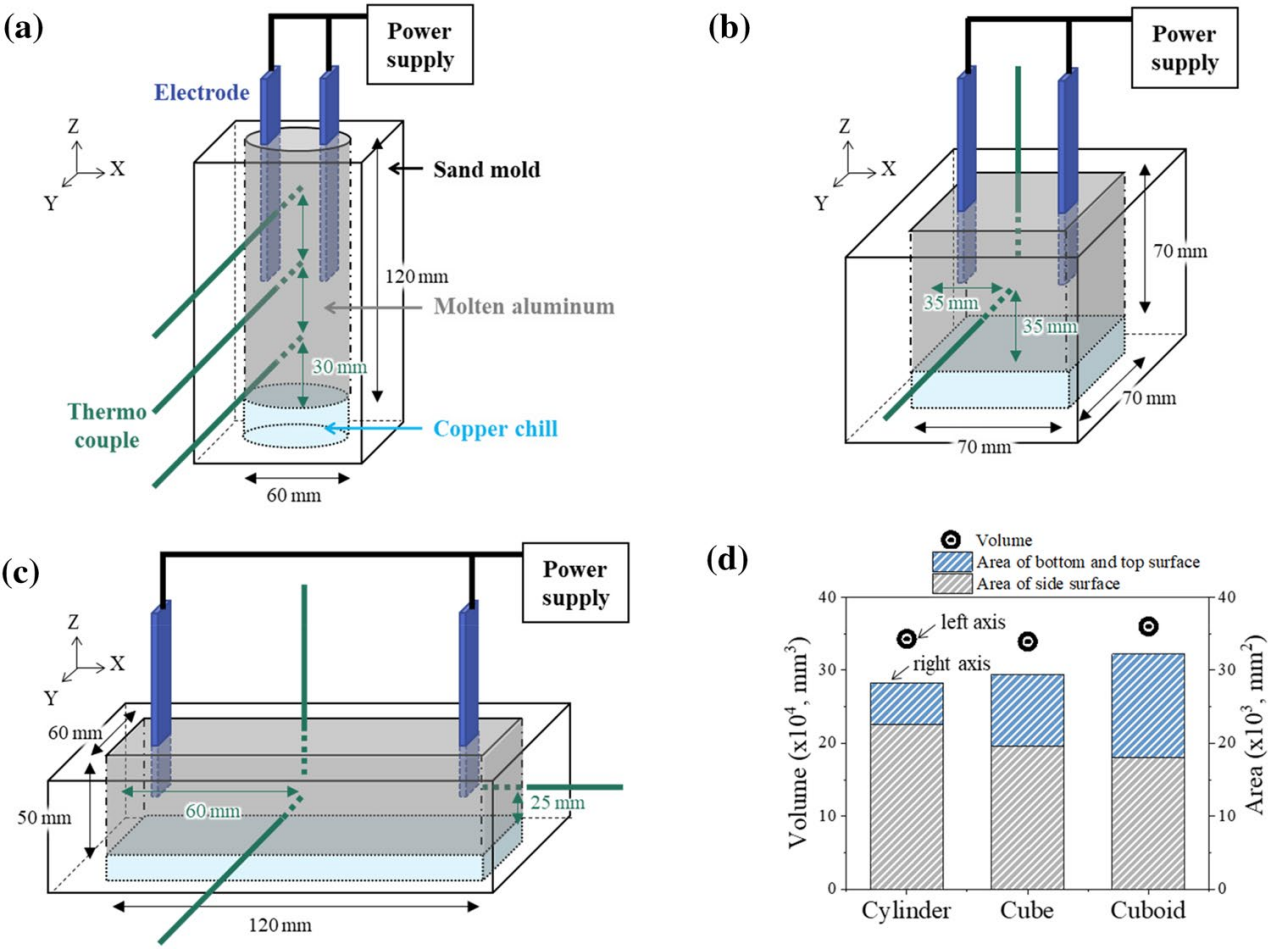
Instrumental setup for (**a**) the cylinder-, (**b**) cube-, and (**c**) cuboid-shaped cavities. (**d**) Volume and surface area of each cavity shape.

Sand molds with three types of cavity shapes, namely, cylinder, cube, and cuboid, were prepared, as shown in Fig. 1a–c. To exclude the effect of the capacity of liquid aluminum during solidification, the inner volume of a cavity is kept almost constant, as shown in Fig. 1d. Each mold has an inner dimension of 60 mm × 120 mm (diameter × height) for the cylinder-, 70 mm × 70 mm × 70 mm (width × depth × height) for the cube-, and 120 mm × 60 mm × 50 mm (width × depth × height) for the cuboid-shaped cavities. In the order of cylinder-, cube-, and cuboid-shaped cavities, the top or bottom area increases, while the side surface area decreased as shown in Fig. 1d. The inner bottom was equipped with a copper plate to achieve directional bottom-up solidification.

Two parallel STS304 electrodes were immersed into liquid aluminum from the top surface at depths of 60, 35, and 25 mm for the cylinder-, cube-, and cuboid-shaped cavities, respectively. The sidewall of the electrode was coated with alumina. The distance between the electrodes was set to 40 mm in the cylinder- and cube-shaped cavities, and 70 mm in the cuboid-shaped cavity. The temperature of liquid aluminum during solidification was recorded using a K-type thermocouple at both the mid- and top heights of the mold in the center of diameter or width of each mold. In the cuboid-shaped cavity, the temperature beside the electrode was additionally measured to analyze the thermogradient effect in the x-direction, as shown in Fig. 1c. The sampling rate of temperature was 100 ms/point, and measured temperature resolution was 0.1 °C with the data logging system (GL240, Graphtech Corporation).

Commercial pure aluminum ingots (1.5 kg) (> 99.7%) were melted in a high-frequency melting furnace using a graphite crucible. When the temperature of the liquid aluminum reached 760 °C, degassing was conducted. After stabilizing for 5 min, liquid aluminum was poured into a sand mold, and electrodes were inserted. The sand mold and electrodes were preheated to 150 °C to prevent the formation of a solid shell from the surface due to rapid solidification. In electrically-assisted solidification (hereafter, EA solidification), when the temperature of liquid aluminum reached 665 °C at the mid-height of the mold, which is near the melting temperature, a direct current of 300 A was applied for 108 s. To reflect the effect of the inserted electrodes in the liquid metal, the electrode was inserted even during solidification without applying electric current, (hereafter, non-EA solidification). All casting experiments were completed in one day to minimize experimental deviations caused by various environmental factors such as mold conditions, environmental temperature, and humidity. For this reason, the number of repetitive experiments was set to two for each experimental condition.

After solidification was completed, the specimen was sectioned longitudinally parallel to the electrodes to observe the macrostructure (YZ plane). The sectioned plan was etched with a solution containing 20 ml HCl, 20 ml HNO3, 20 ml H2O, and 5 ml HF. The fraction of the refined area was analyzed using an open source software ImageJ version 1.53e (available at https://imagej.nih.gov/ij/). To observe the microstructure, specimens were mechanically ground to 1 µm and electrolytically etched using a standard Barker solution at 25 V for 15 min. Five images were analyzed per specimen using a polarization microscope (NICON ECLIPSE MA200), and the grain size was quantitatively measured using image analysis software (IMT i-solution Inc I Solution DT-L).

### Modelling

Numerical analysis was performed using COMSOL Multiphysics 5.0 (COMSOL Inc., USA) to verify the flow phenomenon of liquid pure aluminum to which an electric current was applied. The Navier–Stokes equation was introduced as the governing equation for the liquid pure aluminum flow, and an external force term was added to determine the effect of the electromagnetic force (i.e., Lorentz force). To apply an electromagnetic effect, we also used a generalized form of constitutive relations for the electric and magnetic fields. In addition, an energy conservation equation formulated in terms of temperature was used to identify the electromagnetic heating of liquid aluminum. More details regarding the equations are given in our previous paper (see Supplementary Information)^41^. The top surface of the mold was designated as an open boundary, whereas no slip wall condition was applied to other surfaces. In addition, the convection heat flux condition was applied to realize cooling by the chill at the bottom surface of the mold. For all three types of cavities, the overall computational domain was discretized by free tetrahedral meshes (maximum size of 3 mm, minimum size of 0.03 mm, and maximum growth rate of 1.13), and a numerical study was carried out using a time-dependent solver over a range of 0–15 s.

## Results and discussion

### Solidification behavior considering the cavity shape

**Figure 2:**
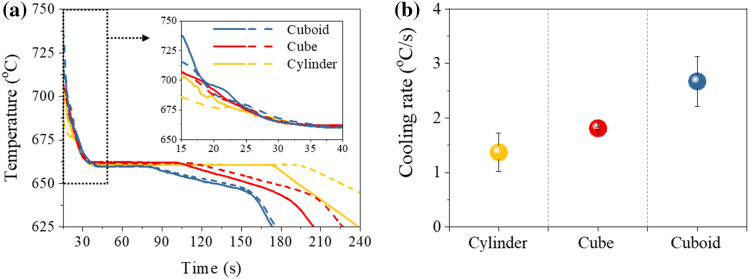
(**a**) Cooling curve measured at the mid-height of the mold, and (**b**) cooling rate of the cylinder-, cube-, and cuboid-shaped cavities without applying electric current during solidification (Non-EA solidification). The solid and dashed line in (**a**) are the results of repeated experiments to confirm the reproducibility.

Cooling curves of non-EA solidification that were measured at the mid-height of the mold for three types of cavity shapes, are presented in Fig. 2a. The liquid metal started to solidify at 660 °C, which is the melting temperature of pure aluminum. In order to evaluate the macroscopic cooling rate after pouring liquid aluminum into each cavity shape, the cooling rate was calculated from the temperature change over 25 s before reaching the melting temperature of 660 °C at the mid-height of the mold. It was measured as 1.7, 2.3 and 2.7 °C/s in the cylinder-, cube-, and cuboid-shaped cavities, respectively (Fig. 2b). The solidification completion time was defined as the period maintained at 660 °C from the initiation to completion of solidification. The larger the bottom or top area and the shorter the height of the mold (Fig. 1d), the faster the cooling rate, and shorter the solidification completion time. The change in solidification completion time according to the cavity shape can be well explained by referring to the modified Chvorinov’s rule^42^, which reflects the difference in shape as well as cavity volume.

**Figure 3:**
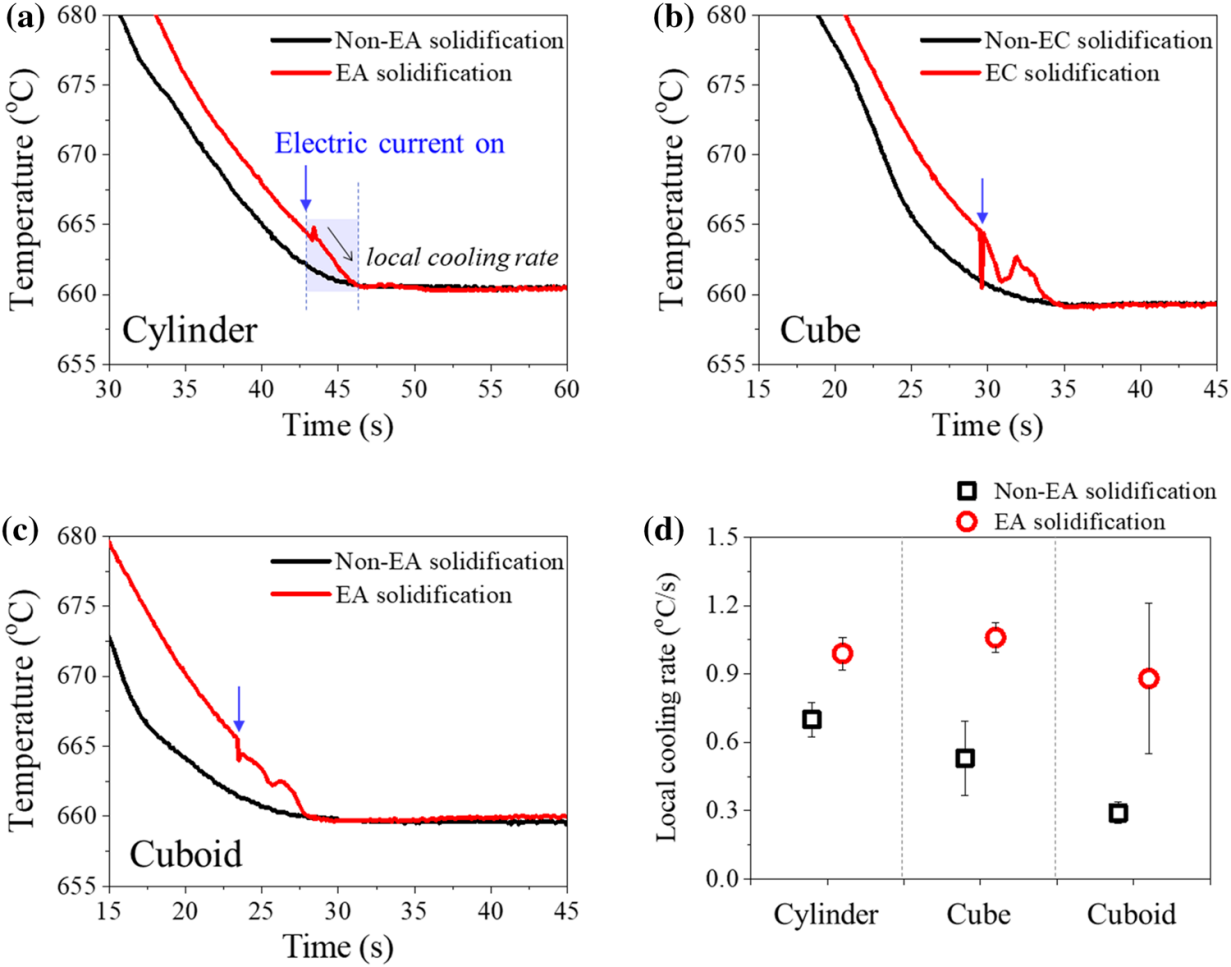
Comparison of cooling curve between non-EA solidification and EA solidification of (**a**) the cylinder-, (**b**) cube-, and (**c**) cuboid-shaped cavities. (**d**) Local cooling rate at each cavity shape.

When an electric current is applied to liquid aluminum, a fluctuation in the cooling curve is observed (Fig. 3a–c). The melting temperature of 660 °C was observed, which is equivalent to non-EA solidification. Here, the cooling rate between 665 °C (the temperature at which the electric current starts to be applied) and 660 °C (melting temperature) was defined as the local cooling rate to compare the effect of electric current on the cooling rate at each cavity shape. Note that local cooling rate is also calculated for non-EA solidification (Fig. 3d), which differed from the macroscopic cooling rate shown in Fig. 2b. The local cooling rate in the EA solidification was higher than that of the non-EA solidification in all three types of cavities (Fig. 3d). In both non-EA and EA solidification, no undercooling was observed in any of the three types of cavities. Note that undercooling was not observed, despite that the local cooling rate increased immediately after the application of an electric current.

### Effect of electric current on grain refinement

**Figure 4:**
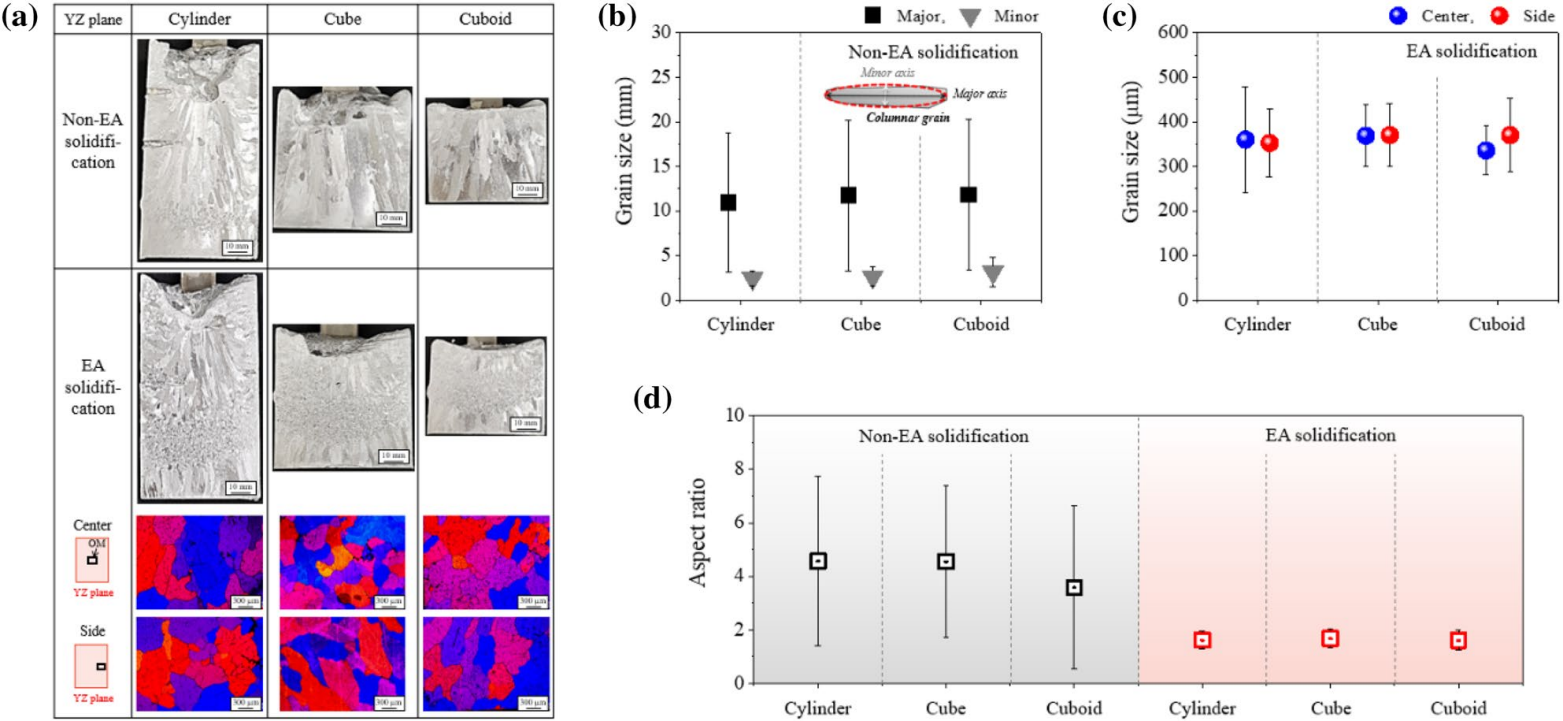
(**a**) Macrostructure and microstructure in the longitudinal area (YZ plane). Average grain size: (**b**) non-EA solidification (major- and minor-axis) and (**c**) EA solidification (in refined area). (**d**) Aspect ratio of grain in non-EA solidification and EA solidification for the cylinder-, cube-, and cuboid-shaped cavities.

The macrostructure of the YZ plane in non-EA and EA solidification is shown in Fig. 4a. In non-EA solidification, coarse columnar grains, which are typical solidification structures, are developed in the cylinder-, cube-, and cuboid-shaped cavities. Since the grains in non-EA solidification are elongated columnar shape, major- and minor-axis of grain were calculated to evaluate the grain size. As shown in Fig. 4b, the average lengths of major- and minor-axis of grain were measured to be ~ 11 mm and ~ 3 mm for all three types of cavities, respectively. The direction of grain growth is almost parallel to the bottom-up direction, due to the directional solidification. In contrast, in EA solidification, equiaxed grains were clearly observed in all three types of cavities. The columnar grains observed in the bottom region during EA solidification solidified before applying an electric current.

In EA solidification, the morphology of refined grains shows an equiaxed shape in both the center and side areas, and the grains are uniformly distributed within the refined zone, as shown in Fig. 4a. In addition, no difference in the grain morphology according to the cavity shape was observed. The grain size in the refined area was measured for both the center and side areas of the YZ plane, and was similar at approximately 350 µm for each cavity (Fig. 4c). Note that the grains are effectively refined as ~ 350 µm in EA solidification, as compared with the grain size of several millimeters measured in non-EA solidification. Also, a lower aspect ratio value of 1.6 ~ 1.7 in EA solidification was confirmed due to the relatively equiaxed shape of grain in all three types of cavity shapes compared to 3.3 ~ 4.6 in non-EA solidification (Fig. 4d).

**Figure 5:**
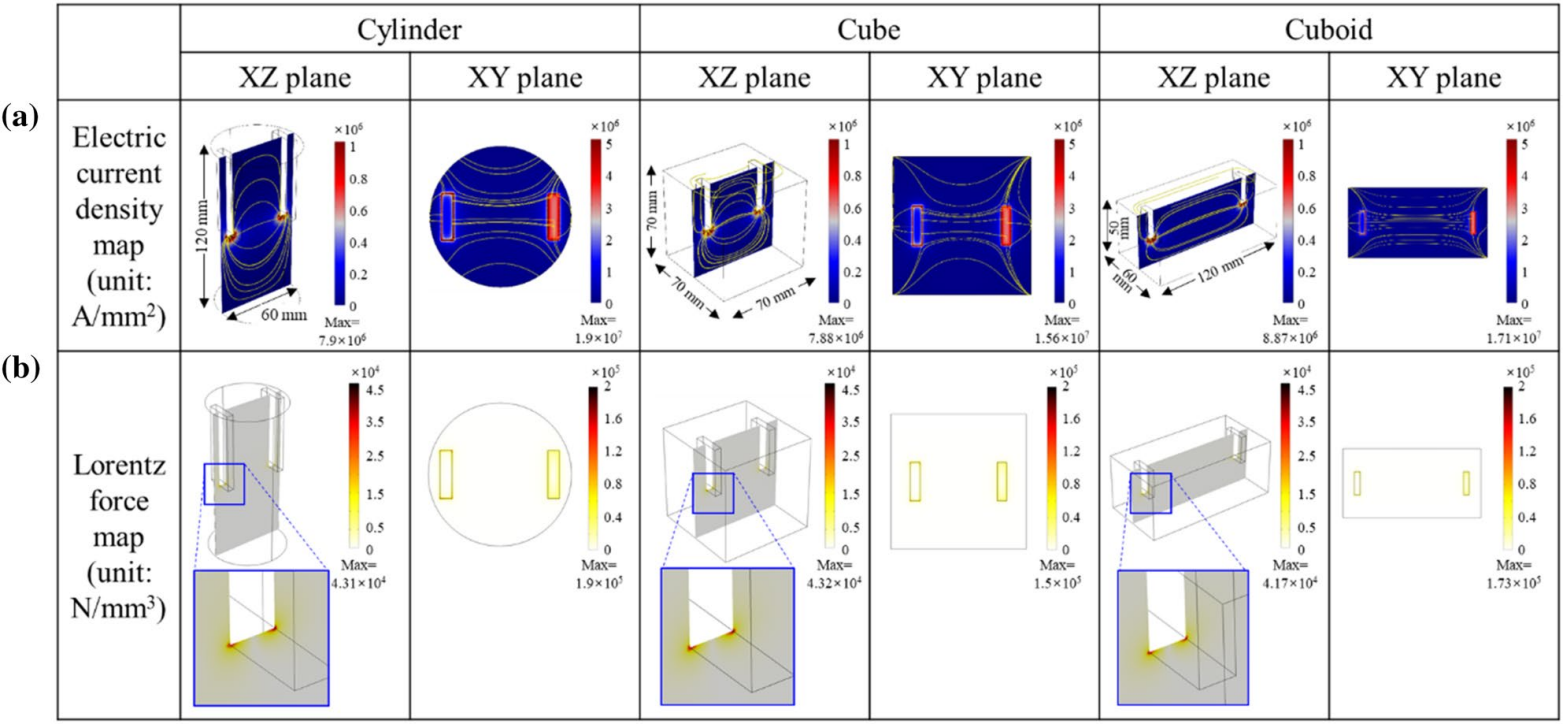
(**a**) Electric current density map and (**b**) Lorentz force map for the cylinder-, cube-, and cuboid-shaped cavities from the numerical simulation.

A numerical simulation was conducted to investigate the effect of the application of an electric current on solidification based on fluid dynamics. When electric current is applied to liquid aluminum, a flow of the density field of electric current is generated in all three types of cavities; in particular, a streamline of the density field of electric current is densely developed in the downward direction of the electrode (Fig. 5a). Between the electrodes, the density field of the electric current was horizontally formed. In the Lorentz force map (Fig. 5b), the intensity of the Lorentz force was the highest below the electrodes. The maximum intensities of the developed Lorentz force in the XY plane are 1.90 × 10^5^, 1.50 × 10^5^, and 1.73 × 10^5^ N/mm^3^ for the cylinder-, cube-, and cuboid-shaped cavities, respectively.

**Figure 6:**
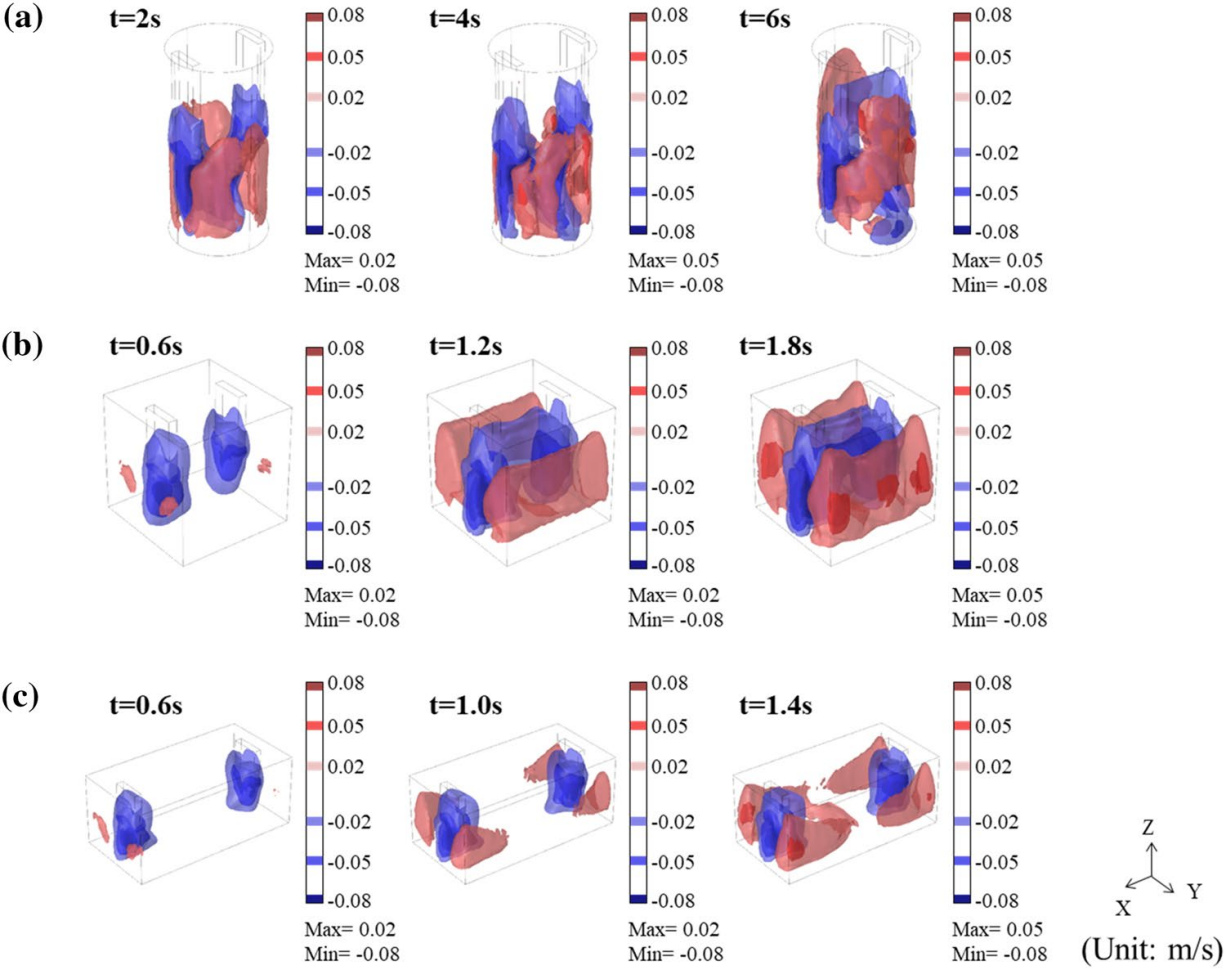
3D velocity map for (**a**) the cylinder-, (**b**) cube-, and (**c**) cuboid-shaped cavities from the numerical simulation.

The 3D velocity maps of the flow in Fig. 6 show a clear circulating flow for all three types of cavities. In previous studies, it was reported that the forced flow of liquid metal occurs by the application of an electric current due to the interaction of electric current, magnetic field, and Lorentz force^28,29,41^. The configuration of the circulating flow depends on the cavity shape. In the cylinder-shaped cavity, the downward flow of liquid aluminum occurs from beneath the electrodes, and the upward flow in a direction rotated 90° from the downward flow. Circulation flow developed in the form of four divisions. (Fig. 6a). For both cube- and cuboid-shaped cavities, a strong downward flow beneath the electrodes was observed. However, a three-layered circulating flow consisting of two upward flows and one downward flow was developed, which were parallel to the XZ plane. In the cuboid-shaped cavity with a longer inter-electrode distance, it takes a longer time to form a three-layered flow than in a cube-shaped cavity with shorter inter-electrode distance. The circulation behavior of the liquid aluminum varies depending on the cavity shape, but the velocities of the upward and downward flows are calculated with similar intensities of 0.05–0.08 m/s for the three types of cavities. The instantaneous increase in local cooling rate by applying an electric current in Fig. 3d can be explained by the acceleration of cooling of liquid aluminum due to the circulating flow. The 2D velocity, temperature, and shear rate maps in the YZ plane for each cavity shape from the numerical simulation with time are described in detail in Figs. S1–3 in Supplementary Information.

**Figure 7:**
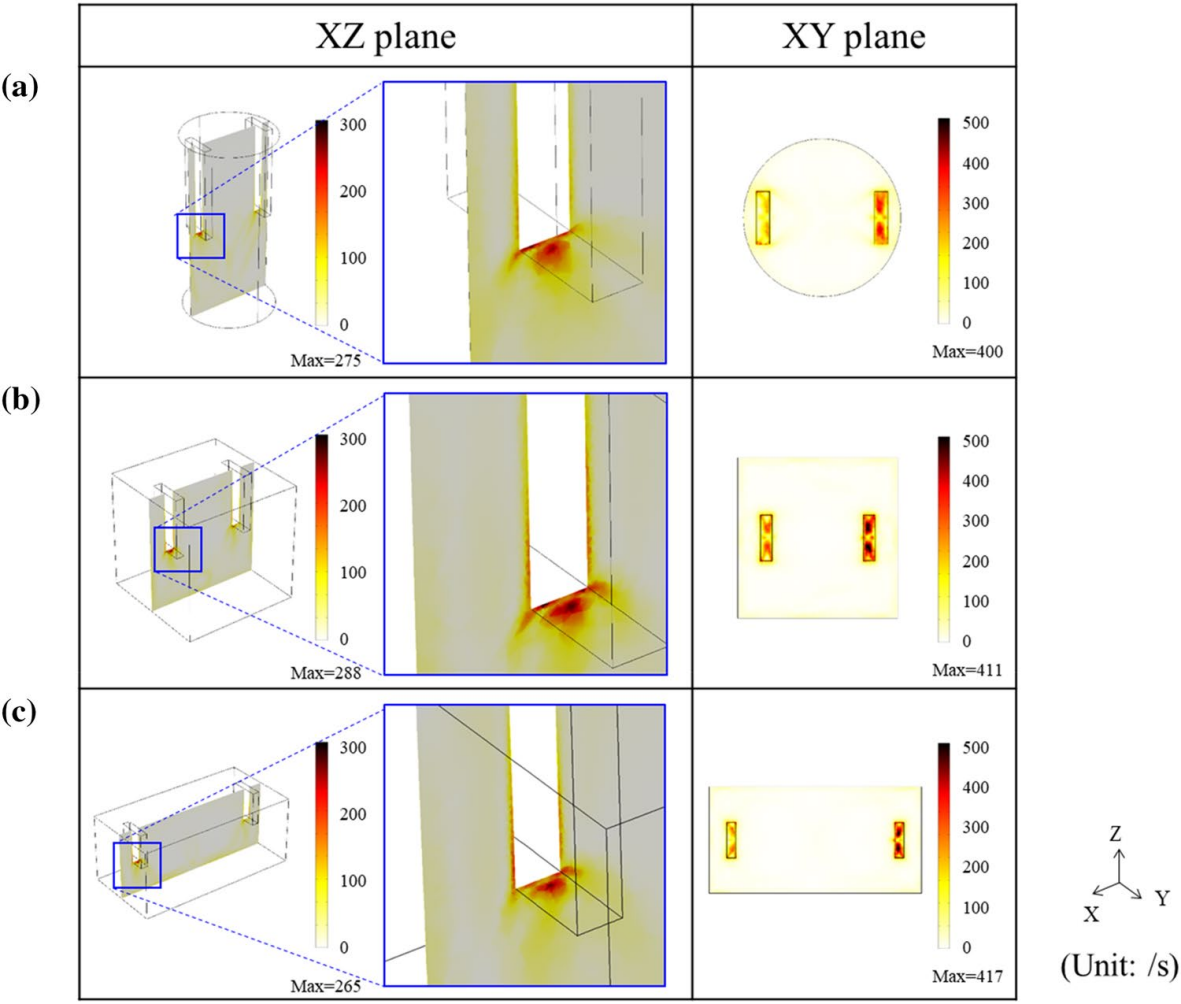
Shear rate map for the cylinder-, cube-, and cuboid-shaped cavities from the numerical simulation.

Because the volume of liquid aluminum and intensity of applied electric current were the same in the three types of cavities, the maximum value of shear rate was similar, approximately 400 s^−1^ for the three types of cavities (Fig. 7). Similar to the Lorentz force, it was confirmed that the intensity of shear rate is the highest below the electrodes. In previous studies on the rheological characteristics of aluminum, a shear rate in the order of hundreds per second is known to induce the breakup of agglomeration of particles, resulting in the modification of the microstructure, including grain (or microparticle) size and morphology^43,44^. Therefore, under selective experimental condition in this study, it is expected that the intensity of the shear rate is sufficient to generate additional nucleation by dendrite fragmentation, and consequently refine the microstructure in EA solidification.

In more detail, in EA solidification, dendrite formed at mold wall can be fragmented and fragmented dendrite tips can be delivered into the unsolidified region due to forced flow of liquid aluminum. These fragmented dendrite tips can act as additional nucleation sites if the size of fragmented dendrite tip is larger than the critical radius of nuclei. In addition, new heterogeneous nucleation can be newly formed in a solid substrate from which nuclei or dendrites are separated by dendrite fragmentation. It can affect increasing the number of nuclei. The increase in the number of nuclei by dendrite fragmentation induced by forced flow of liquid aluminum is expected to have a significant effect on grain refinement in EA solidification compared to non-EA solidification.

Regarding the cavity shape in EA solidification, refined grain size shows similar values at all three types of cavities. The number of nuclei per unit volume can be predicted from the grain size^45^. Therefore, the number of nuclei per unit volume is expected to be similar in all three types of cavities. Referring to Gibbs–Thomson-Ferreira equation for nucleation based on the thermal field gradient^46^, the critical radius (${r}_{c}$) for nonequilibrium homogeneous and heterogeneous nucleation can be given by

1 $$r_{c} = \frac{2}{\Delta T}{\Gamma }$$

where $\Delta T$ is undercooling and ${\Gamma }$ is Gibbs–Thomson coefficient. Gibbs–Thomson coefficient can be expressed as

2 $${\Gamma }\left( {\vec{r}} \right) = \nabla {\mathbf{T}} \cdot \hat{n}A\left( {\vec{r}} \right)$$

where $\vec{r}$ is the vector radius, $\nabla {\mathbf{T}}$ is the thermal gradient normal to the surface area of $A\left( {\vec{r}} \right)$, and $\hat{n}$ is the unit normal vector^46^. Especially for $\nabla {\mathbf{T}}$,

3 $$\nabla {\mathbf{T}} = \left[ {\frac{\partial T}{{\partial V}} \cdot \nabla V + \frac{\partial T}{{\partial P}} \cdot \nabla P + \mathop \sum \limits_{i = 1}^{n - 1} \frac{\partial T}{{\partial C_{i} }} \cdot \nabla C_{i} + \nabla T} \right]$$

where $$V, P, C_{i}$$ and $$T$$ are volume, pressure, species, and temperature^46^. Since all three cavity shapes have similar cavity volumes, the same composition as pure aluminum, and the same pressure condition (~ 1 atm), it can be assumed that $$\nabla {\mathbf{T}}$$ is dominantly related to $$\nabla T$$. In our experiment, cuboid-shaped cavity has larger surface area than a cylinder-shaped cavity, so temperature gradient in cuboid-shaped cavity is the smallest among three types of cavities. Therefore, thermal gradient in cuboid-shaped cavity is also expected to be the smallest among three types of cavity shape. It means the critical radius of nuclei in cuboid-shape cavity is the smallest, while it is the largest in cylinder-shaped cavity ($${r}_{critical, cuboid}< {r}_{critical, cube}<{r}_{critical, cylinder})$$. Nuclei larger than the critical radius of nuclei ($${r}_{critical}$$) in each cavity shape will survive and grow into crystal grains. Also, the smaller the size of critical radius of nuclei, the higher the number of surviving nuclei for grain growth among various size of fragmented dendrite tips. Therefore, the number of effective nuclei is expected to be the highest in cuboid-shaped cavity, whereas the lowest in cylinder-shaped cavity. Especially, in cylinder-shaped cavity, some nuclei may be remelted due to the relatively long dwell time at the melting temperature. Therefore, the total number of effective nuclei per time ($${\raise0.7ex\hbox{${N_{{nuclei}} }$} \!\mathord{\left/ {\vphantom {{N_{{nuclei}} } t}}\right.\kern-\nulldelimiterspace} \!\lower0.7ex\hbox{$t$}}$$) is expected to be: cuboid-shaped cavity > cube-shaped cavity > cylinder-shaped cavity. However, solidified unit volume per time ($${\raise0.7ex\hbox{${V_{{unit}} }$} \!\mathord{\left/ {\vphantom {{V_{{unit}} } t}}\right.\kern-\nulldelimiterspace} \!\lower0.7ex\hbox{$t$}}$$) is the highest in cuboid-shaped cavity, while it is the lowest in cylinder-shaped cavity due to the difference in cooling rate. Finally, the number of nuclei per unit volume ($${\raise0.7ex\hbox{${N_{{nuclei}} }$} \!\mathord{\left/ {\vphantom {{N_{{nuclei}} } {V_{{unit}} }}}\right.\kern-\nulldelimiterspace} \!\lower0.7ex\hbox{${V_{{unit}} }$}}$$) can be derived by considering $${\raise0.7ex\hbox{${N_{{nuclei}} }$} \!\mathord{\left/ {\vphantom {{N_{{nuclei}} } t}}\right.\kern-\nulldelimiterspace} \!\lower0.7ex\hbox{$t$}}$$ and $${\raise0.7ex\hbox{${V_{{unit}} }$} \!\mathord{\left/ {\vphantom {{V_{{unit}} } t}}\right.\kern-\nulldelimiterspace} \!\lower0.7ex\hbox{$t$}}$$, and this value is expected to be similar for all three types of cavities as shown in Fig. S4. The similar $${\raise0.7ex\hbox{${N_{{nuclei}} }$} \!\mathord{\left/ {\vphantom {{N_{{nuclei}} } {V_{{unit}} }}}\right.\kern-\nulldelimiterspace} \!\lower0.7ex\hbox{${V_{{unit}} }$}}$$ is expected to have a major influence on the similar grain size in all three types of cavity shapes in EA solidification.

As shown in Fig. 6, although the form of the circulating flow was different depending on the cavity shape, liquid aluminum mixed well within the inner cavity of the mold. Therefore, the new nuclei can be dispersed uniformly in the unsolidified region, resulting in a small variation in the grain size in the refined zone for each cavity shape. For all three types of cavities, the circulating flow of liquid aluminum in the region at the top height of the mold is inhibited because of gravity, which reduces the shear rate. The observed refined zone located at the mid-height of the mold is explained by both bottom-up solidification and the reduced shear rate at the top height of the mold.

**Figure 8:**
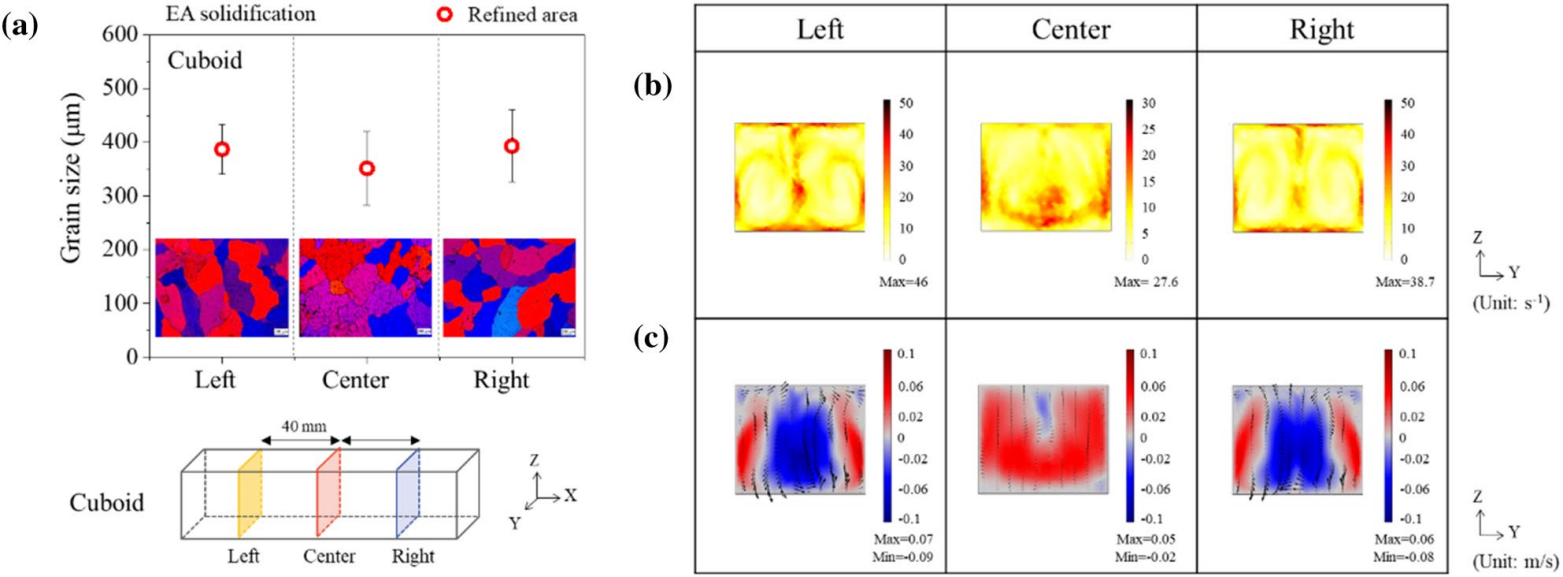
(**a**) Grain size in refined area of YZ plane at cuboid shaped cavity. Numerical simulation: (**b**) 2D shear rate map and (**c**) 2D velocity map after 4 s of applying electric current at cuboid-shaped cavity.

In the cuboid-shaped cavity with the longest inter-electrode distance among three cavity shapes, the grain size was approximately 390 μm in each section of the YZ plane of the left, and right regions (as described in Fig. 8a). In Fig. 8b, the maximum shear rate was observed to be approximately 46, 27.6, and 38.7 s^−1^ for the left, center, and right regions of the YZ plane, respectively. It is in the order of 10 lower than the shear rate formed in the region below the electrodes (~ 400 s^−1^). Therefore, in the inter-electrode region, rather than new nuclei generated by shear rate, the nuclei can be delivered to the inter-electrode region by the circulating flow, resulting in a uniform grain size within the refined zone despite the long inter-electrode distance.

### Effect of electric current on fraction of grain refinement

**Figure 9:**
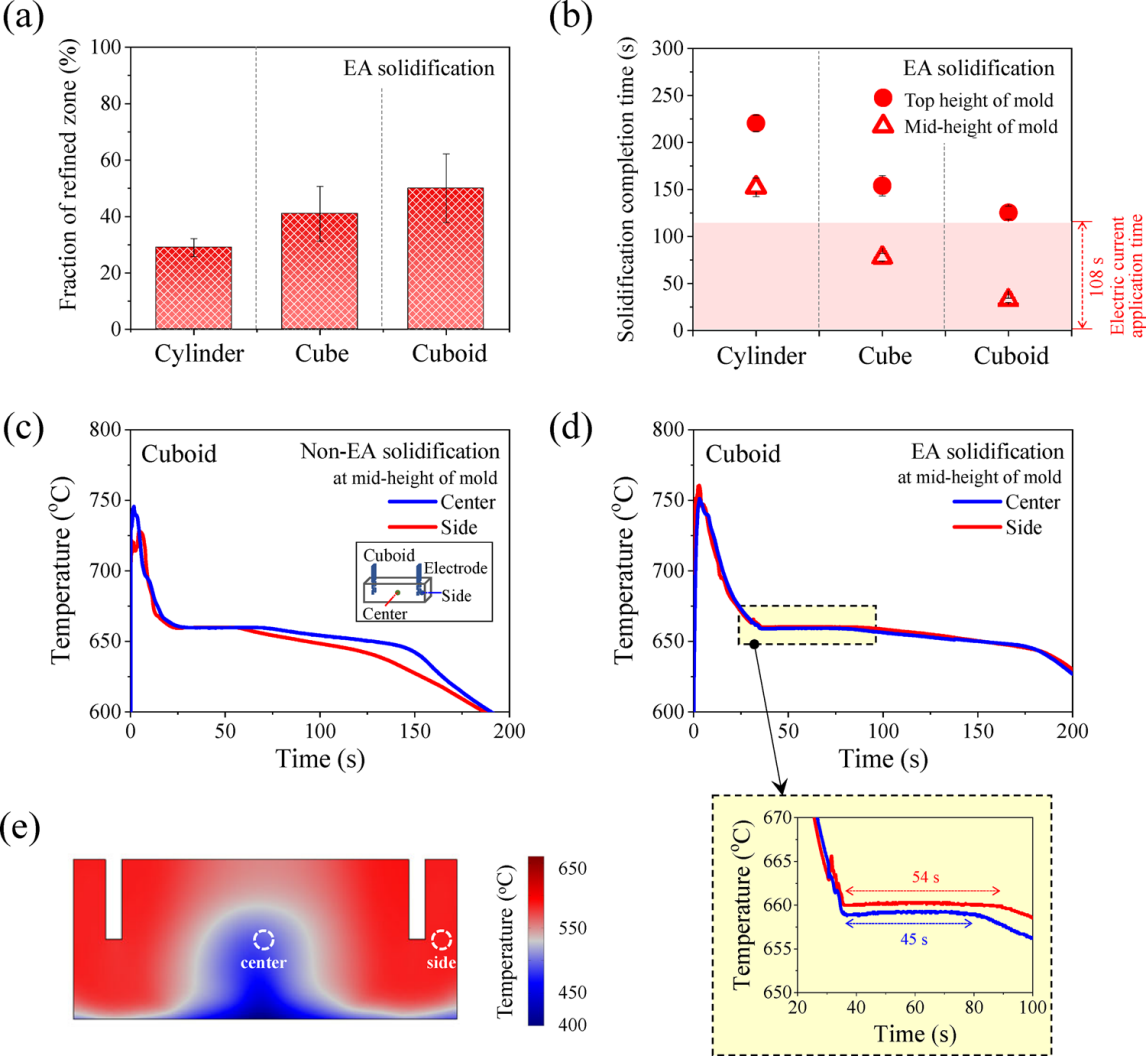
(**a**) Fraction of the refined zone in the longitudinal YZ plane in EA solidification. (**b**) Solidification time at the mid- and top height of the mold in EA solidification for the cylinder-, cube-, and cuboid-shaped cavities. Measured cooling curve at the mid-height of the cuboid-shaped cavity in (**c**) non-EA solidification and (**d**) EA solidification. (**e**) Numerically calculated 2D temperature distribution map of XZ plane in cuboid-shaped cavity.

**Figure 10:**
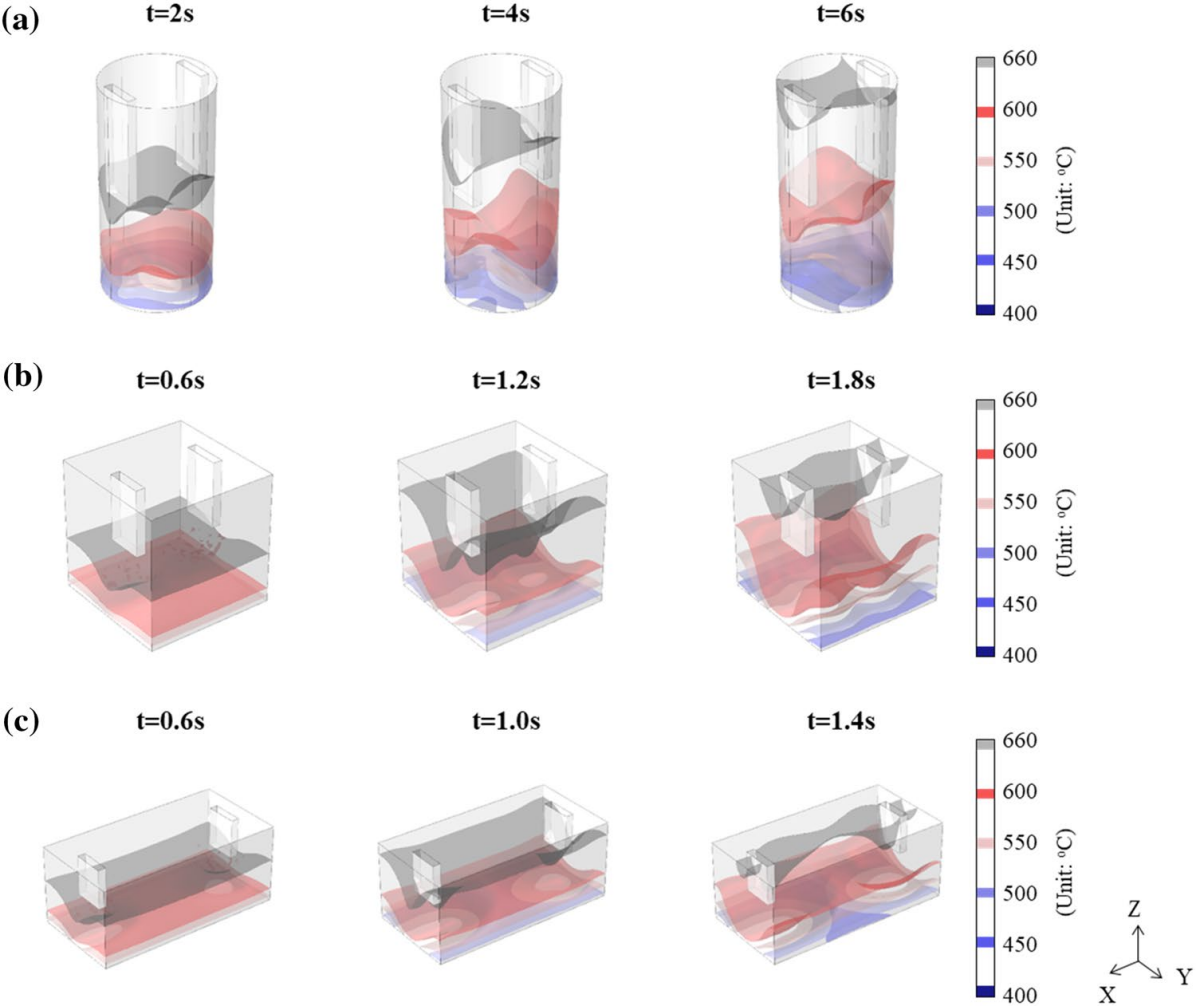
3D temperature distribution map of the liquid aluminum from the numerical simulation for (**a**) the cylinder-, (**b**) cube-, and (**c**) cuboid-shaped cavities.

The area fraction of the refined zone is expected to be affected by the relative relationship between the electric current application time and solidification completion time. The area fractions of the refined zone were approximately 29, 41, and 50% for the cylinder-, cube-, and cuboid-shaped cavities, respectively (Fig. 9a). The area fraction of the grain refined zone was calculated based on the longitudinal 2D YZ plane shown in Fig. 4a. In the case of the cylinder-shaped cavity, an electric current is removed (108 s) before the solidification at the mid-height of the mold is complete (Fig. 9b). Therefore, after removing the electric current, it is expected that the grain growth of existing nuclei will occur in the unsolidified region without an additional supply of nuclei by dendrite fragmentation. In the cube-shaped cavity, the electric current application time was approximately 20 s longer than the solidification completion time at the mid-height of the mold (Fig. 9b). This implies that additional nuclei can be generated in the unsolidified liquid aluminum above the mid-height of the mold, inducing a higher fraction of the refined zone as compared to the cylinder-shaped cavity. In both cylinder- and cube- shaped cavities, the refined zone in the YZ plane has a V-shape as shown in Fig. 4a. This can be explained by the V-shaped distribution of temperature in the YZ plane, owing to the circulating flow in the 3D temperature distribution map of liquid aluminum (Fig. 10a and b).

In the cuboid-shaped cavity, an electric current was applied until the completion of solidification at the top height of the mold, as shown in Fig. 9b. In the 3D temperature distribution map of liquid aluminum in the cuboid-shaped cavity (Fig. 10c), the liquid aluminum in the center region of the width cools faster than the liquid aluminum next to the electrodes. Therefore, even after the liquid aluminum at mid-height of mold is solidified, nuclei that is newly generated by applying an electric current in the unsolidified liquid aluminum present under the electrodes can be supplied to the top height of mold in the center of the width, resulting in the highest fraction of refined zone. The measured temperatures in the center and side areas at the mid-height of the mold are shown in Fig. 9c and d. In non-EA solidification, solidification is completed faster in the side region, which is a heat dissipation path. However, in EA solidification, solidification in the center of the width is completed more than 9 s earlier than on the side region at the mid-height of the mold. This matches well with the simulation results for the 2D temperature distribution map of XZ plane (Fig. 9e). The V-shaped refined zone shown in the macrostructure (Fig. 4a) may also be affected by the V-shaped distribution of temperature in the YZ plane, as shown in Fig. 10c.

## Conclusions

In this study, we used an electric current as an external energy source to obtain refined grains in the as-cast state. This study highlights the effect of electric current on solidification behavior with different cavity shapes in a fixed-volume. The solidification structure was remarkably refined to approximately 350 μm from several millimeters of grain size (~ 11 mm), and equiaxed grains with lower aspect ratio are uniformly distributed with a small deviation of grain size in the refined zone, by applying an electric current during solidification in the cylinder-, cube-, and cuboid-shaped cavities. In the numerical simulation, the occurrence of circulating flow was observed, and the form of circulating flow in liquid aluminum depends on the cavity shape. In addition, it was confirmed that the Lorentz force generated by the interaction between electric current and liquid metal showed similar values in each cavity shape, and the shear rate was also similar as 400 s^−1^ for the three types of cavities, which is expected to be sufficient to induce the breaking up of agglomeration of particles and modify the microstructure. It is expected that new nuclei may be generated by a strong shear rate when unsolidified aluminum passes under the electrode, and it can be delivered to the unsolidified region, owing to the circulating flow. The observation of similar grain size in EA solidification with different cavity shape is expected to be influenced by the nucleation behavior and solidification according to the cavity shape. The fraction of the refined zone varied depending on the cavity shape, and the lowest was approximately 29% in the cylinder-shaped cavity, and the highest was approximately 50% in the cuboid-shaped cavity. This is explained by the relative relationship between the solidification completion time and the electric current application time.

The use of electric current as a process parameter to obtain a refined microstructure is an emerging technology. Because the shape of a real product is complex, there are many considerations to control the microstructure of a cast product. By applying an electric current to the liquid metal, the circulating flow is developed, and it depends on the cavity shape and electrical conditions. Therefore, to effectively control the microstructure using electric current, the electric current-induced phenomenon related to nucleation and solidification should be carefully considered. This study can provide insight into the microstructural control considering various cavity shapes using electric current-assisted solidification.

## Supplementary Information

Supplementary Information.

## Data Availability

All data included in this study are available upon request by contact with the corresponding author.
