# Supplementary material for: Effect of cavity shape on microstructural evolution of pure aluminum in electrically-assisted solidification
Source: Sci Rep. 2023 Feb 28;13:3382. doi: 10.1038/s41598-023-29522-y (PMC9975195; doi:10.1038/s41598-023-29522-y)
Supplement: Supplementary file 1 — Supplementary Information. [file 41598_2023_29522_MOESM1_ESM.docx]

**Supplementary Information**

***Constitutive equations for the numerical analysis to to evaluate the effect of the electric current on the pure aluminum melt using COMSOL Multiphysics***

Electric current: Generalized form of the constitutive relations for the electric field.

$\mathbf{J}=\left(\sigma+\varepsilon_{0}\varepsilon_{r}\frac{\partial}{\partial t}\right)\mathbf{E}$ (1)

where **J** is the current density field, $\sigma$ is the electrical conductivity, $\varepsilon_{0}$ is the vacuum permittivity, $\varepsilon_{r}$ is the relative permittivity, and **E** is the electric field.

Magnetic field: Generalized form of the constitutive relation for the magnetic field.

$\sigma\frac{\partial\mathbf{A}}{\partial t}+\nabla\times\left(\mu_{0}^{-1}\mu_{r}^{-1}\mathbf{B}\right)-\sigma\mathbf{v}\times\mathbf{B}=\mathbf{J}'$ with $\mathbf{B}=\nabla\times\mathbf{A}$ (2)

where **A** is the magnetic vector potential, **B** is the magnetic flux densitiy, $\mu_{0}$ is the vacuum permeability, and $\mu_{r}$ is the relative permeability.

Volumetric flow of the pure aluminum melt: Navier-Stokes equation considering with the incompressibility condition ($\nabla\cdot\mathbf{u}=0$) and the electromagnetic force (i.e. Lorentz force).

$\rho\left[\frac{\partial\mathbf{u}}{\partial t}+\left(\mathbf{u}\times\nabla\right)\mathbf{u}\right]=-\nabla p+\eta\nabla^{2}\mathbf{u}+\mathbf{J}\times\mathbf{B}+\mathbf{F}_{g}$ (3)

where $\rho$ is the density, **u** is the velocity field, *p* is the pressure, $\eta$ is the dynamic viscosity, and $\mathbf{F}_{g}$ is the gravitational force density.

Heat transfer in the melt: Energy conservation equation formulated in terms of temperature.

$\rho C_{P}\left(\frac{\partial T}{\partial t}+\mathbf{u}\cdot\nabla T\right)=\nabla\cdot\left(k\nabla T\right)+Q$ (4)

where $C_{P}$ is the heat capacity at constant pressure, *T* is the absolute temperature, *k* is the thermal conductivity, and *Q* is heat source induced by the electromagnetic Joule heating.

Cooling through the bottom wall: Convective heat flux on the lower surface of the domain/

$q_{0}=h\cdot\left(T_{\text{ext}}-T\right)$ (5)

where $q_{0}$ is the convective heat flux, *h* is the heat transfer coefficient of the copper, and$T_{\text{ext}}$ is the external temperature.


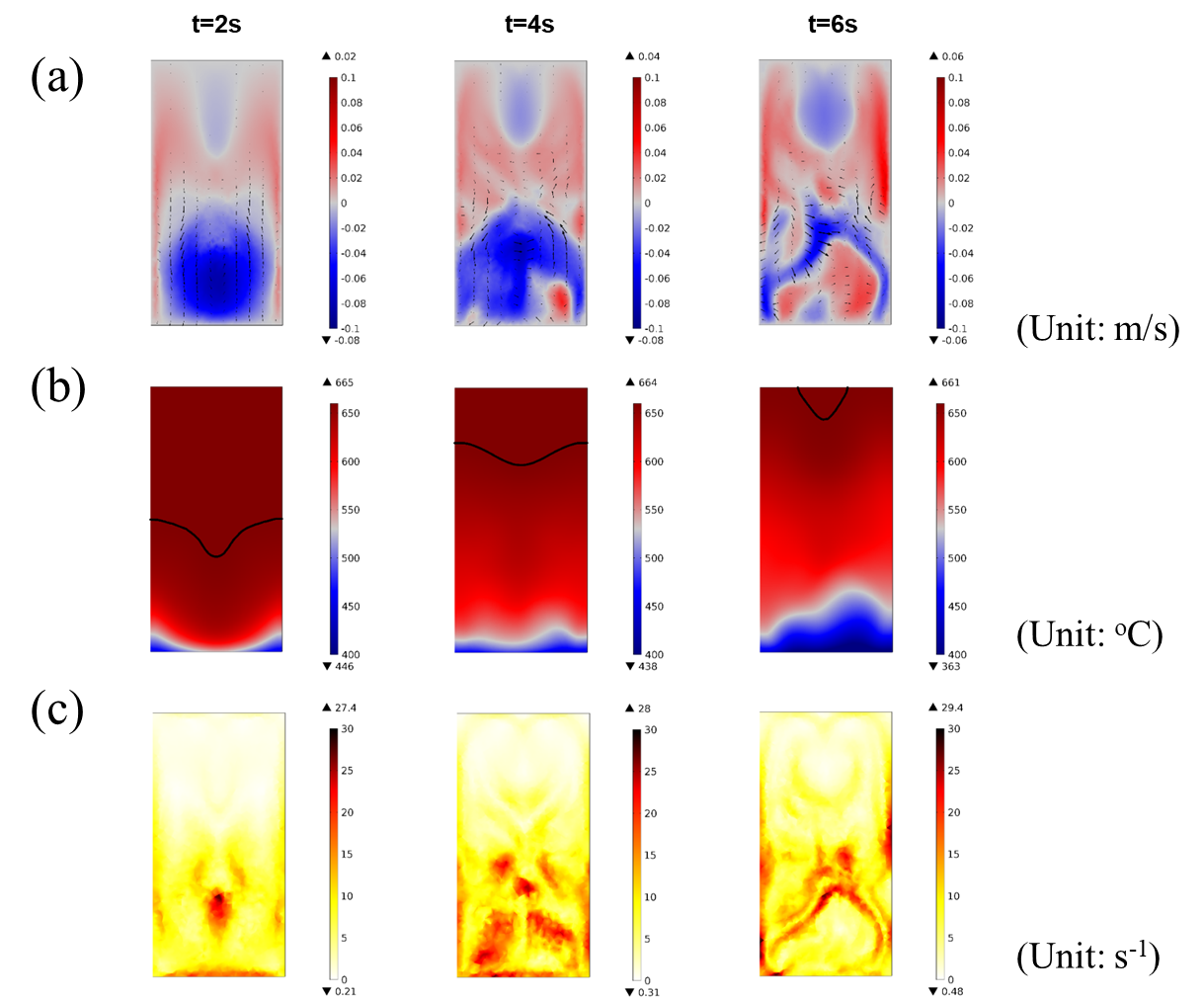


Figure S1. (a) 2D velocity map, (b) 2D temperature map, (c) 2D shear rate map in YZ plane for cylinder-shaped cavity from numerical simulation.


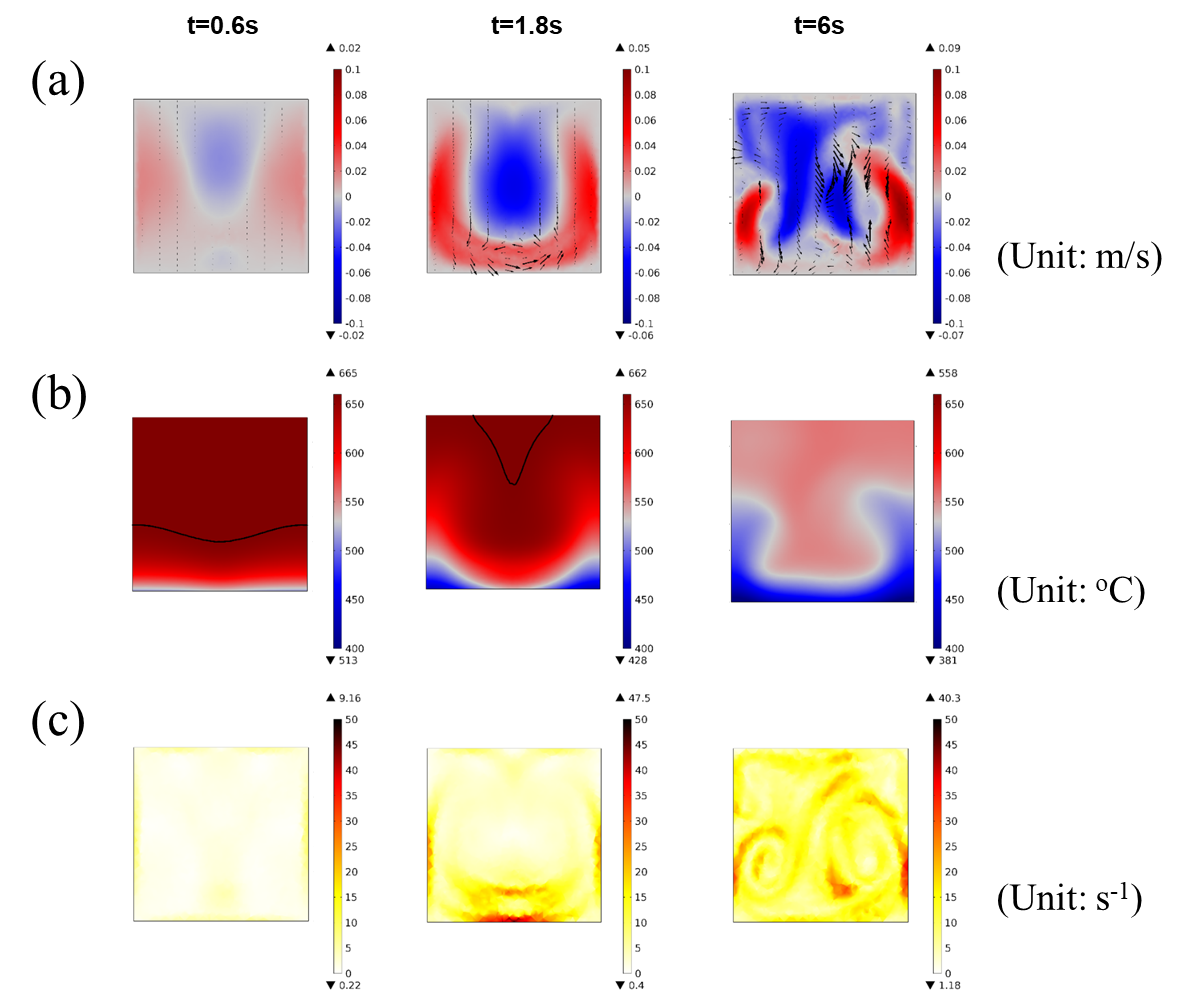


Figure S2. (a) 2D velocity map, (b) 2D temperature map, (c) 2D shear rate map in YZ plane for cube-shaped cavity from numerical simulation.


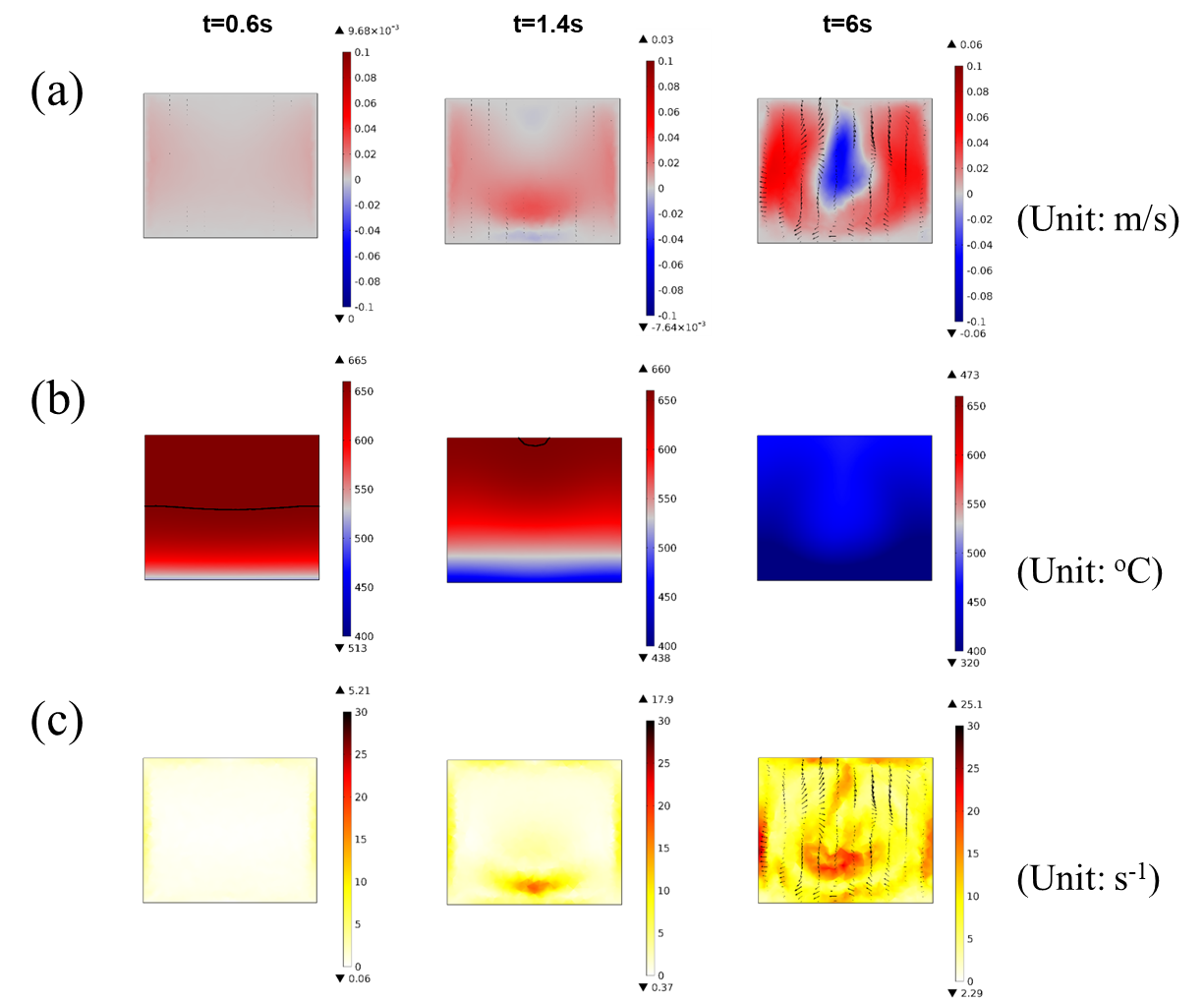


Figure S3. (a) 2D velocity map, (b) 2D temperature map, (c) 2D shear rate map in YZ plane for cuboid-shaped cavity from numerical simulation.


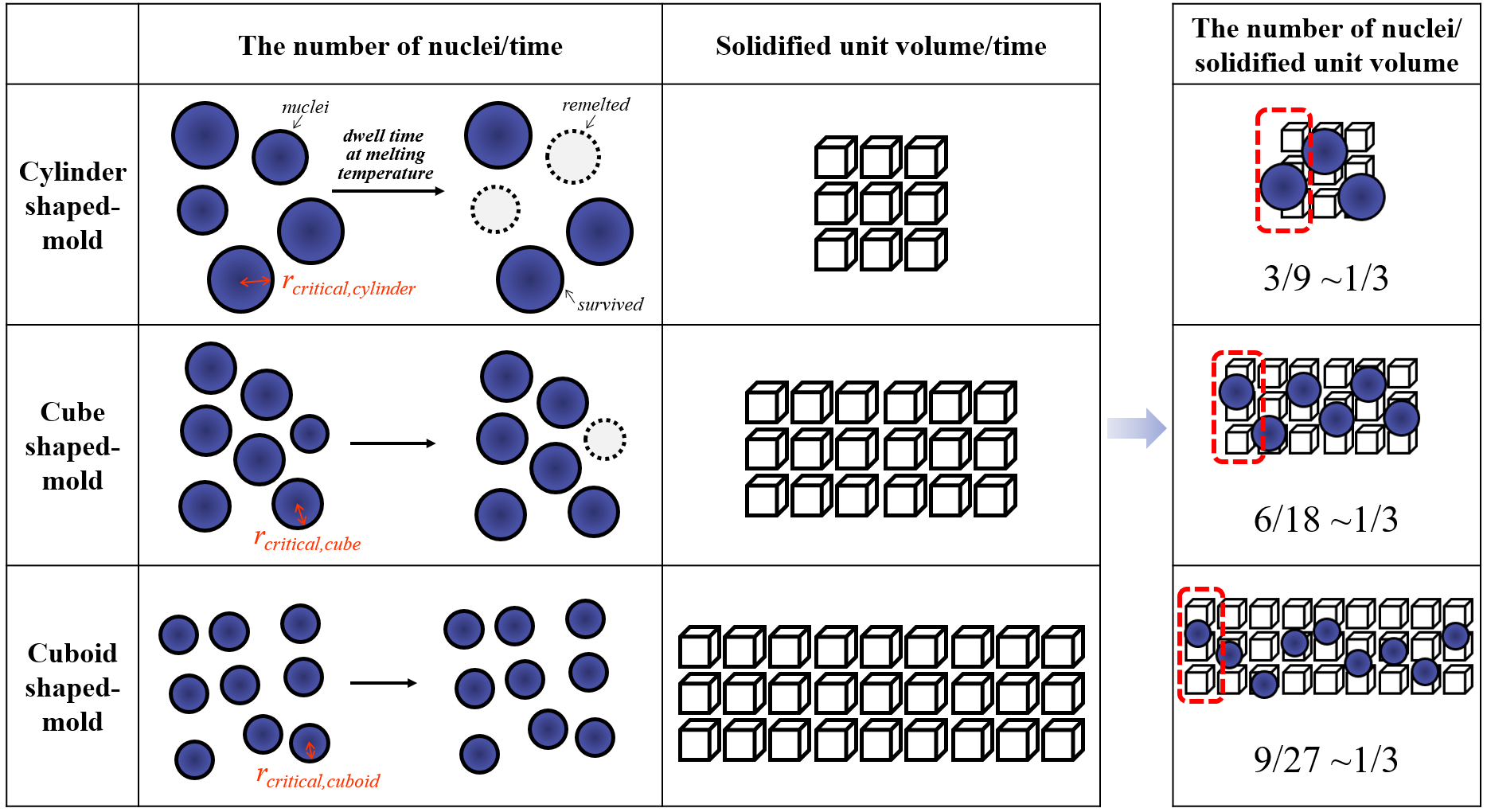


Figure S4. Schematic diagram describing the number of nuclei per unit volume according to cavity shape. The critical radius of nuclei is the smallest in cuboid-shaped cavity and the largest in cylinder-shaped cavity ($r_{critical, cuboid}< r_{critical, cube}<r_{critical, cylinder}$).
